# Antituberculosis: Synthesis and Antimycobacterial Activity of Novel Benzimidazole Derivatives

**DOI:** 10.1155/2013/926309

**Published:** 2013-12-05

**Authors:** Yeong Keng Yoon, Mohamed Ashraf Ali, Tan Soo Choon, Rusli Ismail, Ang Chee Wei, Raju Suresh Kumar, Hasnah Osman, Farzana Beevi

**Affiliations:** ^1^Institute for Research in Molecular Medicine, Universiti Sains Malaysia, 11800 Minden, Penang, Malaysia; ^2^New Drug Discovery Research, Department of Medicinal Chemistry, Alwar Pharmacy College, Alwar, Rajasthan 301030, India; ^3^New Drug Discovery Research, Department of Medicinal Chemistry, Sunrise University, Alwar, Rajasthan 301030, India; ^4^Centre of Excellence for Research in AIDS (CERiA), University of Malaya, 50603 Kuala Lumpur, Malaysia; ^5^School of Chemical Science, Universiti Sains Malaysia, 11800 Minden, Penang, Malaysia; ^6^Department of Chemistry, College of Science, King Saud University, P.O. Box 2455, Riyadh, Saudi Arabia

## Abstract

A total of seven novel benzimidazoles were synthesized by a 4-step reaction starting from 4-fluoro-3-nitrobenzoic acid under relatively mild reaction conditions. The synthesized compounds were screened for their antimycobacterial activity against *M. tuberculosis* H_37_Rv (MTB-H_37_Rv) and INH-resistant *M. tuberculosis* (INHR-MTB) strains using agar dilution method. Three of them displayed good activity with MIC of less than 0.2 **μ**M. Compound ethyl 1-(2-(4-(4-(ethoxycarbonyl)-2-aminophenyl)piperazin-1-yl)ethyl)-2-(4-(5-(4-fluorophenyl)pyridin-3-ylphenyl-1H-benzo[d]imidazole-5-carboxylate (**5g**) was found to be the most active with MIC of 0.112 **μ**M against MTB-H_37_Rv and 6.12 **μ**M against INHR-MTB, respectively.

## 1. Introduction

Tuberculosis (TB) is the oldest documented infectious disease. It is the only disease which does not require any vector for transportation from one person to another [1]. The primary site of infection is the lungs, followed by dissemination via the circulatory and lymphatic system to secondary sites including the bones, joints, liver, and spleen. 

In 2010, there were 8.8 million (range: 8.5–9.2 million) incident cases of TB, 1.1 million (range: 0.9–1.2 million) deaths from TB among HIV-negative people, and an additional 0.35 million (range: 0.32–0.39 million) deaths from HIV-associated TB [2]. The introduction of the first-line drugs like streptomycin, para-aminosalicylic acid, and isoniazid for treatment some 50 years ago has witnessed a remarkable decline in TB cases all over the world. The active TB is currently treated with a four-first-line-drug regimen comprising mainly isoniazid, rifampicin, pyrazinamide, and ethambutol for a period of at least 6 months [3, 4]. However, the disease has been undergoing a resurgence in the last two decades driven by variety of changes in social, medical, and economic factors as well as *M. tuberculosis* resistance to the aforementioned drugs itself. 

The resurgence of TB is now one of the most serious public health concerns worldwide. Despite its global impact on world health, TB is considered a neglected disease, and no new anti-TB therapeutics have been introduced into the market over the last half-century. The last drug with a new mechanism of action approved (rifampicin) was discovered in 1963 [5]. Therefore there is an urgent need for development of new drug leads to combat this chronic infectious disease.

The benzimidazole nucleus is of significant importance in medicinal chemistry research, and many benzimidazole-containing compounds exhibit important biological properties such as antiviral [6], anti-inflammatory [7], and anti-HIV [8]. In the light of the affinity they display towards a variety of enzymes and protein receptors, medicinal chemists thus classify them as “*privileged substructures*” for drug design [9].

Recently, there has been reported work done on utilizing benzimidazole derivatives to counter TB with relatively good results [10–12], thus further reinforcing our belief that benzimidazole could potentially be a lead compound in our effort to discover new potent anti-TB agents. In the present paper, we wish to report the synthesis and antimycobacterial activity of novel 2-substituted benzimidazole derivatives.

## 2. Materials and Methods

### 2.1. Chemistry

All chemicals were supplied by Sigma-Aldrich (USA) and Merck Chemicals (Germany). Purity of the compounds was checked on thin layer chromatography (TLC) plates (silica gel G) in the solvent system chloroform-methanol (9 : 1). The spots were located under short (254 nm)/long (365 nm) UV light. Elemental analyses were performed on Perkin Elmer 2400 Series II CHN Elemental Analyzer and were within ±0.4% of the calculated values. Physical properties and elemental analysis results of compounds (Table 1). ^1^H and ^13^C NMR were performed on Bruker Avance 300 (^1^H: 300 MHz, ^13^C: 75 MHz) spectrometer in CDCl_3_ using TMS as internal standard (Table 2). Mass spectra were recorded on Varian 320-MS TQ LC/MS using ESI.

#### 2.1.1. Procedure for the Preparation of *Ethyl-4-fluoro-3-nitrobenzoate* ****(****1****)

4-Fluoro-3-nitrobenzoic acid (5 g, 27 mmol) was refluxed in ethanol (50 mL) and concentrated H_2_SO_4_ (2 mL) for 8 hours. After completion of reaction (as evident from TLC), the solvent was evaporated under reduced pressure. The aqueous layer was extracted with ethyl acetate (25 mL × 3). The organic layer was dried over Na_2_SO_4_ and concentrated under reduced pressure to yield **1** as cream-coloured powder (75%).

#### 2.1.2. Procedure for the Preparation of Ethyl 3-amino-4-(4-(2-((4-(ethoxycarbonyl)-2-nitrophenyl)amino)ethyl)piperazin-1-yl)benzoate**    **(****2****)

Ethyl-4-fluoro-3-nitrobenzoate, **1** (0.5 g, 2.34 mmol), N-(2-aminoethyl)piperazine (0.15 mL, 1.16 mmol), and N,N-diisopropylethylamine (DIPEA) (0.49 mL, 2.78 mmol) were mixed in dichloromethane (10 mL). The reaction mixture was stirred overnight at room temperature. After completion of reaction (as evident from TLC), the reaction mixture was washed with water (10 mL × 2) followed by 10% Na_2_CO_3_ solution (10 mL). The organic layer was dried over Na_2_SO_4_ and concentrated under reduced pressure to afford **2** as brown oil (91%).

#### 2.1.3. Procedure for the Preparation of Ethyl 3-amino-4-(4-(2-((2-amino-4-(ethoxycarbonyl)phenyl)amino)ethyl)piperazin-1-yl)benzoate**    **(****3****)

Ethyl 3-amino-4-(4-(2-((4-(ethoxycarbonyl)-2-nitrophenyl)amino)ethyl)piperazin-1-yl)benzoate, **2** (0.486 g, 1 mmol), ammonium formate (0.378 g, 6 mmol), and Pd/C (50 mg) were mixed in ethanol (10 mL). The reaction mixture was refluxed until completion (solution turned colourless). The reaction mixture was then filtered through Celite 545. The filtrate was evaporated under reduced pressure. It was resuspended in ethyl acetate and washed with water, dried over Na_2_SO_4_, and evaporated to dryness to yield **3** (70%).

#### 2.1.4. General Procedure for the Preparation of Sodium Bisulfite Adducts of 4-Substituted Benzaldehyde****(****4a–g****)

Appropriate benzaldehyde (10 mmol) was dissolved in ethanol (20 mL). Sodium metabisulfite (15 mmol) in 5 mL water was added in portion over 5 minutes. The reaction mixture was stirred at room temperature for 1 hour and subsequently stirred at 4°C overnight. The precipitate formed was filtered and dried to afford sodium bisulfite adducts (55%–90%).

#### 2.1.5. General Procedure for the Preparation of 2-Substituted Benzimidazole Derivatives**    **(****5a–g****)

Ethyl  3-amino-4-(4-(2-((2-amino-4-(ethoxycarbonyl)phenyl)amino)ethyl)piperazin-1-yl)benzoate, **3** (1 mmol) and various sodium bisulfite adducts, **4** (1.5 mmol) were dissolved in DMF (5 mL). The reaction mixture was stirred at 90°C under N_2_ atmosphere for 24–48 hours. After completion of reaction (evident by TLC), the reaction mixture was diluted in ethyl acetate (25 mL) and washed with water (10 mL × 3). The organic layer was collected, dried over Na_2_SO_4_, and evaporated under reduced pressure to afford compounds **5a–g** in 63–90% yields.

### 2.2. Biology

*In vitro* antimycobacterial activity of the compounds was evaluated against *Mycobacterium tuberculosis *H_37_Rv (MTB-H_37_Rv) using the broth dilution method as reported by Collins and Franzblau [13]. The minimum inhibitory concentration (MIC) values were determined using *M. tuberculosis* (MTB-H_37_Rv) and INH-resistant *M. tuberculosis* (INHR-MTB) strains. The MIC was defined as the minimum concentration of compound required to inhibit 90% of bacterial growth.

## 3. Results and Discussion

### 3.1. Chemistry

**Scheme 1:**
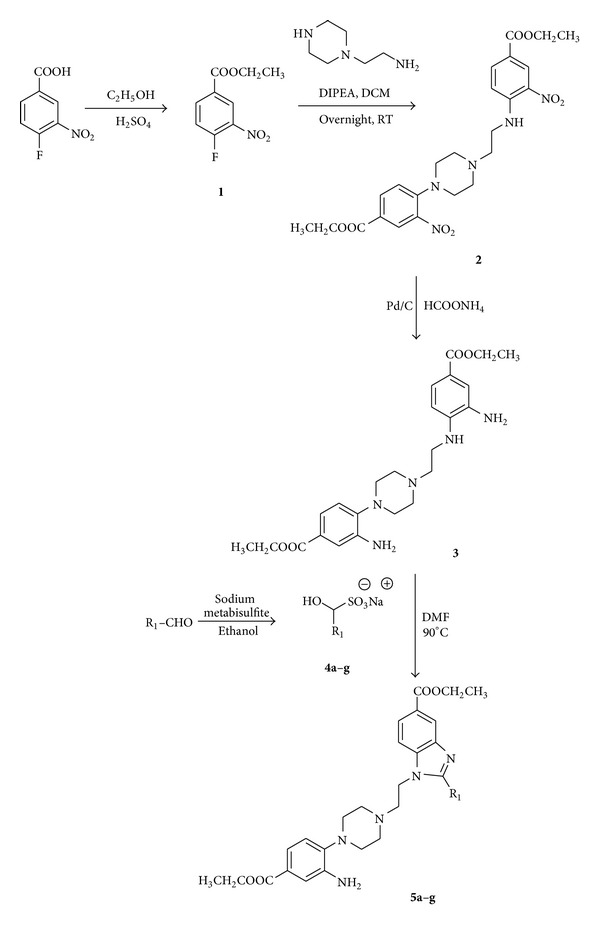
Protocol for synthesis of **5a–g**.

Our synthetic study into novel benzimidazoles started with 4-fluoro-3-nitro benzoic acid which was esterified in the presence of catalytic sulfuric acid in ethanol by refluxing for 8 hours to afford ethyl-4-fluoro-3-nitrobenzoate** 1**, in 75% yield. The ethylbenzoate **1** was then treated with amine and DIPEA in dry dichloromethane at room temperature to yield ethyl 3-amino-4-(4-(2-((4-(ethoxycarbonyl)-2-nitrophenyl)amino)ethyl)piperazin-1-yl)benzoate **2**, which was then reduced to **3** using ammonium formate and 10% Pd/C for 1 hour to give 70% yield. The phenylenediamine **3** was then refluxed with various substituted bisulfite adducts of aromatic aldehydes [14] in DMF overnight to afford benzimidazole derivatives **5a–g** in moderate to good yields (63–90%). The structure of the novel benzimidazoles was confirmed by chromatographic and spectroscopic analysis. The mechanism for the formation of the novel benzimidazole derivatives is proposed and summarized in Scheme 1.

### 3.2. Pharmacology

A total of seven novel benzimidazole derivatives were synthesized and then analyzed for their antimycobacterial activities against *Mycobacterium tuberculosis *H_37_Rv (MTB-H_37_Rv) and INH-resistant *M. tuberculosis* (INHR-MTB). Results are shown in Table 3 with standard drug isoniazid as comparison.

We synthesized compounds with a wide range of substitution including compounds with electron-donating as well as electron-withdrawing groups. Generally, we found that electron-withdrawing group substituents at 4-position in the phenyl ring are important for good activities. Three compounds (**5b**, **5d**, and **5g**) showed excellent potency against MTB-H_37_Rv with MIC of <0.2 *μ*M. Of all 9 compounds which have been tested, compound **5g **was found to be the most active with MIC of 0.112 *μ*M against MTB-H_37_Rv and 6.12 *μ*M against INHR-MTB. This was followed by **5d** (MIC = 0.135 *μ*M against MTB-H_37_Rv and 16.25 *μ*M against INHR-MTB) and **5b** (MIC = 0.195 *μ*M against MTB-H_37_Rv and 24.12 *μ*M 6.12 against INHR-MTB). However the electron-donating groups such as 2,4-dihydroxyl and 4-methoxyl substituted analogue compounds produced moderate inhibitory activity against MTB *Mycobacterium tuberculosis.* This clearly showed that the presence of electron-withdrawing group, especially halogen substitution at 4-position of the phenyl ring, caused marked improvement in antimycobacterial activity.

All the compounds were also tested for cytotoxicity (IC_50_) in VERO cells at a concentration of 62.5 *μ*g/mL. After 72 h of exposure, viability was assessed on the basis of cellular conversion of MTT into a formazan product using the Promega CellTiter 96 nonradioactive cell proliferation assay according to the manufacturer's protocol. All of the active compounds were found to be nontoxic till 62.5 *μ*g/mL.

## 4. Conclusion

In conclusion, we have synthesized successfully a series of novel benzimidazole derivatives with good antimycobacterial properties against *M. tuberculosis*. Encouraged by the positive results we have reported here, further modification on the 4-position on the bisulfite adducts as well as quantitative structure-activity relationship (QSAR) is currently in progress in our laboratory. It is conceivable that derivatives showing antimycobacterial activity can be further modified to exhibit better potency than standard drugs.

## Supplementary Material

S1: ^1^H NMR for compound 5d.
S2: ^13^C NMR for compound 5d.
S3: Direct infusion LC-MS for compound 5d.

## Figures and Tables

**Table 1:** Physical properties and analytical results of compounds **5a**–**g**.

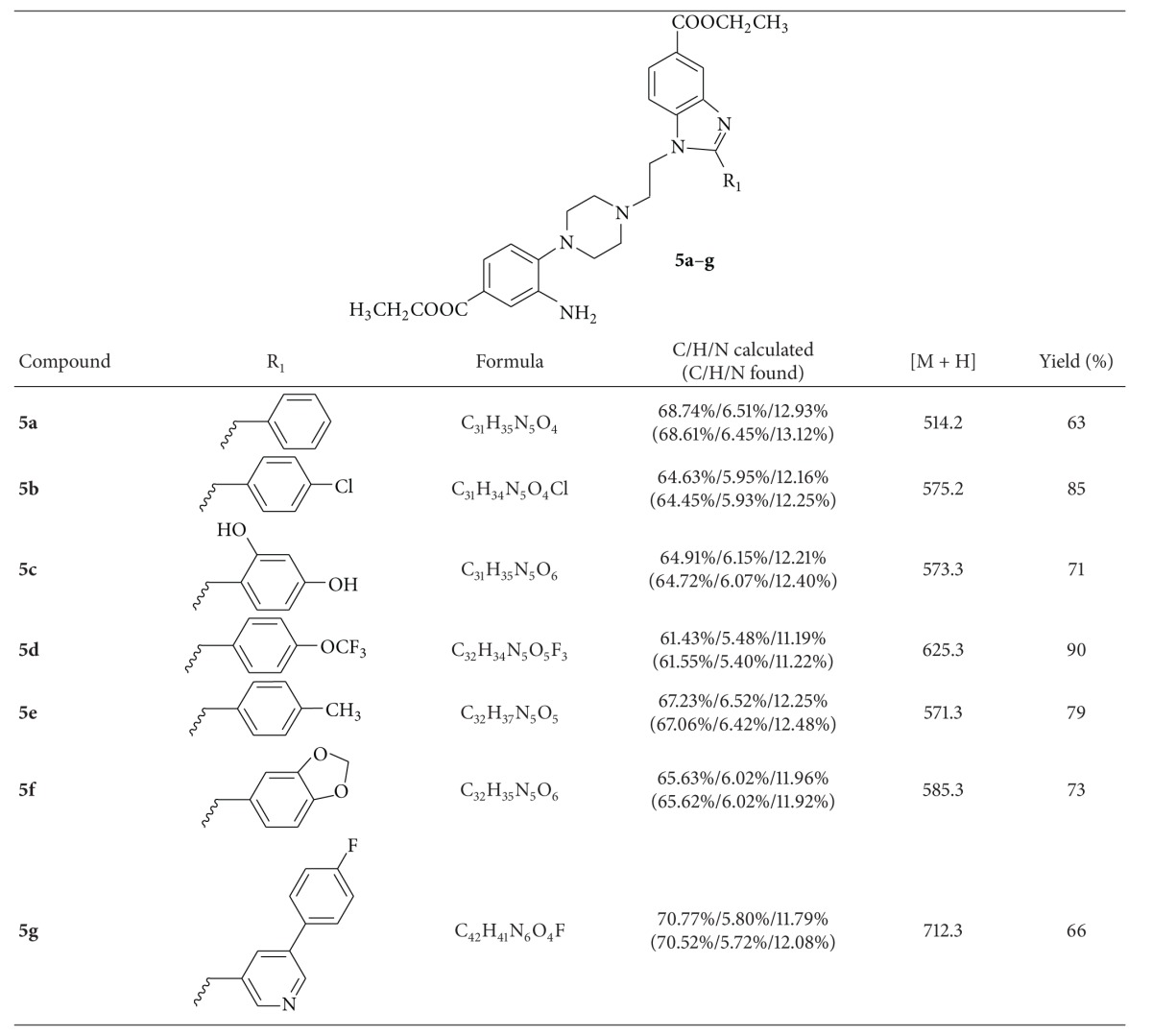

**Table 2:** ^
1^H NMR results for compounds **1**, **2**, and **5a**–**g** and selected ^13^C NMR results.

Compound	NMR (*δ* ppm)
Ethyl-4-fluoro-3-nitrobenzoate, **1**	^ 1^H NMR: 1.44 (3H, t, *J* = 7.2 Hz), 4.45 (2H, q, *J* = 7.2 Hz), 7.41 (1H, d, *J* = 8.4 Hz), 8.70 (1H, dd, *J*_1_ = 8.4 Hz, *J*_2_ = 1.5 Hz), 8.89 (1H, s)

Ethyl 3-amino-4-(4-(2-((4-(ethoxycarbonyl)-2-nitrophenyl)amino)ethyl)piperazin-1-yl)benzoate, **2**	^ 1^H NMR: 1.38 (3H, t, *J* = 7.2 Hz), 1.43 (3H, t, *J* = 7.2 Hz), 2.79 (4H, t, *J* = 4.8 Hz), 2.90 (2H, t, *J* = 6.9 Hz), 3.29 (4H, t, *J* = 4.8 Hz), 3.52 (2H, q, *J* = 7.2 Hz), 4.37 (2H, t, *J* = 6.9 Hz), 4.44 (2H, q, *J* = 7.2 Hz), 6.87 (1H, d, *J* = 8.4 Hz), 7.11 (1H, d, *J* = 8.4 Hz), 8.15 (1H, dd, *J*_1_ = 8.4 Hz, *J*_2_ = 1.5 Hz), 8.33 (1H, dd, *J*_1_ = 8.4 Hz, *J*_2_ = 1.5 Hz), 8.74 (1H, s), 8.84 (1H, s)

Ethyl 1-(2-(4-(4-(ethoxycarbonyl)-2-aminophenyl)piperazin-1-yl)ethyl)-2-phenyl-1H-benzo[d]imidazole-5-carboxylate, **5a**	^ 1^H NMR: 1.37 (3H, t, *J* = 7.2 Hz), 1.44 (3H, t, *J* = 7.2 Hz), 2.53 (4H, t, *J* = 4.8 Hz), 2.85 (2H, t, *J* = 6.9 Hz), 3.91 (4H, t, *J* = 4.8 Hz), 4.35 (2H, q, *J* = 7.2 Hz), 4.45 (2H, t, *J* = 6.9 Hz), 4.46 (2H, q, *J* = 7.2 Hz), 6.90–7.20 (5H, m), 7.50 (1H, d, *J* = 8.4 Hz), 8.11 (1H, dd, *J*_1_ = 8.4 Hz, *J*_2_ = 1.5 Hz), 8.57 (1H, s)

Ethyl 1-(2-(4-(4-(ethoxycarbonyl)-2-aminophenyl)piperazin-1-yl)ethyl)-2-(4-chlorophenyl)-1H-benzo[d]imidazole-5-carboxylate, **5b**	^ 1^H NMR: 1.38 (3H, t, *J* = 7.2 Hz), 1.46 (3H, t, *J* = 7.2 Hz), 2.54 (4H, t, *J* = 4.8 Hz), 2.86 (2H, t, *J* = 6.9 Hz), 3.91 (4H, t, *J* = 4.8 Hz), 4.35 (2H, q, *J* = 7.2 Hz), 4.45 (2H, t, *J* = 6.9 Hz), 4.46 (2H, q, *J* = 7.2 Hz), 6.92 (1H, d, *J* = 8.4 Hz), 7.40 (1H, s), 7.47 (1H, dd, *J*_1_ = 8.4 Hz, *J*_2_ = 1.5 Hz), 7.52 (1H, d, *J* = 8.4 Hz), 7.65 (2H, d, *J* = 8.4 Hz), 7.85 (2H, d, *J* = 8.4 Hz), 8.12 (1H, dd, *J*_1_ = 8.4 Hz, *J*_2_ = 1.5 Hz), 8.58 (1H, s)

Ethyl 1-(2-(4-(4-(ethoxycarbonyl)-2-aminophenyl)piperazin-1-yl)ethyl)-2-(2, 4-dihydroxyphenyl)-1H-benzo[d]imidazole-5-carboxylate, **5c**	^ 1^H NMR: 1.37 (3H, t, *J* = 7.2 Hz), 1.44 (3H, t, *J* = 7.2 Hz), 2.53 (4H, t, *J* = 4.8 Hz), 2.85 (2H, t, *J* = 6.9 Hz), 3.91 (4H, t, *J* = 4.8 Hz), 4.35 (2H, q, *J* = 7.2 Hz), 4.44 (2H, t, *J* = 6.9 Hz), 4.45 (2H, q, *J* = 7.2 Hz), 6.68 (1H, s), 6.87 (1H, dd, *J*_1_= 8.4 Hz, *J*_2_ = 1.5 Hz), 6.92 (1H, d, *J* = 8.4 Hz), 7.40 (1H, s), 7.44 (1H, d, *J* = 8.4 Hz), 7.50 (1H, d, *J* = 8.4 Hz), 7.56 (1H, dd, *J*_1_ = 8.4 Hz, *J*_2_ = 1.5 Hz), 8.16 (1H, dd, *J*_1_ = 8.4 Hz, *J*_2_ = 1.5 Hz), 8.56 (1H, s)

Ethyl 1-(2-(4-(4-(ethoxycarbonyl)-2-aminophenyl)piperazin-1-yl)ethyl)-2-(4-(trifluoromethoxy)phenyl)-1H-benzo[d]imidazole-5-carboxylate, **5d**	^ 1^H NMR: 1.38 (3H, t, *J* = 7.2 Hz), 1.46 (3H, t, *J* = 7.2 Hz), 2.53 (4H, t, *J* = 4.8 Hz), 2.85 (2H, t, *J* = 6.9 Hz), 3.91 (4H, t, *J* = 4.8 Hz), 4.35 (2H, q, *J* = 7.2 Hz), 4.45 (2H, t, *J* = 6.9 Hz), 4.46 (2H, q, *J* = 7.2 Hz), 6.92 (1H, d, *J* = 8.4 Hz), 7.40 (1H, s), 7.47 (1H, dd, *J*_1_ = 8.4 Hz, *J*_2_ = 1.5 Hz), 7.52 (1H, d, *J* = 8.4 Hz), 7.66 (2H, d, *J* = 8.4 Hz), 7.84 (2H, d, *J* = 8.4 Hz), 8.12 (1H, dd, *J*_1_ = 8.4 Hz, *J*_2_ = 1.5 Hz), 8.59 (1H, s) ^13^C NMR: 14.78, 43.48, 50.46, 54.47, 57.53, 61.08, 61.41, 110.28, 114.00, 116.46, 119.19, 120.97, 122.20, 125.92, 126.19, 126.23, 126.72, 134.17, 139.17, 141.08, 143.09, 143.32, 154.48, 160.05, 167.13, 167.36

Ethyl 1-(2-(4-(4-(ethoxycarbonyl)-2-aminophenyl)piperazin-1-yl)ethyl)-2-p-tolyl-1H-benzo[d]imidazole-5-carboxylate, **5e**	^ 1^H NMR: 1.37 (3H, t, *J* = 7.2 Hz), 1.45 (3H, t, *J* = 7.2 Hz), 2.53 (4H, t, *J* = 4.8 Hz), 2.85 (2H, t, *J* = 6.9 Hz), 3.91 (4H, t, *J* = 4.8 Hz), 4.35 (2H, q, *J* = 7.2 Hz), 4.45 (2H, t, *J* = 6.9 Hz), 4.46 (2H, q, *J* = 7.2 Hz), 4.48 (3H, s), 6.90 (1H, d, *J* = 8.4 Hz), 7.40 (1H, s), 7.46 (1H, dd, *J*_1_ = 8.4 Hz, *J*_2_ = 1.5 Hz), 7.52 (1H, d, *J* = 8.4 Hz), 7.66 (2H, d, *J* = 8.4 Hz), 7.84 (2H, d, *J* = 8.4 Hz), 8.12 (1H, dd, *J*_1_ = 8.4 Hz, *J*_2_ = 1.5 Hz), 8.57 (1H, s)

Ethyl 1-(2-(4-(4-(ethoxycarbonyl)-2-aminophenyl)piperazin-1-yl)ethyl)-2-(benzo[d][1, 3]dioxol-5-yl)-1H-benzo[d]imidazole-5-carboxylate, **5f**	^ 1^H NMR: 1.38 (3H, t, *J* = 7.2 Hz), 1.46 (3H, t, *J* = 7.2 Hz), 2.53 (4H, t, *J* = 4.8 Hz), 2.85 (2H, t, *J* = 6.9 Hz), 3.90 (4H, t, *J* = 4.8 Hz), 4.35 (2H, q, *J* = 7.2 Hz), 4.45 (2H, t, *J* = 6.9 Hz), 4.46 (2H, q, *J* = 7.2 Hz), 6.10 (2H, s), 6.92 (1H, d, *J* = 8.4 Hz), 7.40 (1H, s), 7.47 (1H, dd, *J*_1_ = 8.4 Hz, *J*_2_ = 1.5 Hz), 7.35 (1H, d, *J* = 8.4 Hz), 7.45–7.55 (3H, m), 8.08 (1H, dd, *J*_1_ = 8.4 Hz, *J*_2_ = 1.5 Hz), 8.55 (1H, s)

Ethyl 1-(2-(4-(4-(ethoxycarbonyl)-2-aminophenyl)piperazin-1-yl)ethyl)-2-(4-(5-(4-fluorophenyl)pyridin-3-yl)phenyl)-1H-benzo[d]imidazole-5-carboxylate, **5g**	^ 1^H NMR: 1.39 (3H, t, *J* = 7.2 Hz), 1.47 (3H, t, *J* = 7.2 Hz), 2.54 (4H, t, *J* = 4.8 Hz), 2.85 (2H, t, *J* = 6.9 Hz), 3.93 (4H, t, *J* = 4.8 Hz), 4.32 (2H, q, *J* = 7.2 Hz), 4.45 (2H, t, *J* = 6.9 Hz), 4.46 (2H, q, *J* = 7.2 Hz), 6.90–8.20 (10H, m), 8.59 (1H, s), 8.70 (2H, s)

**Table 3:** Antimycobacterial activity of compounds **5a–g** against *M. tuberculosis* H_37_Rv and INH-resistant *Mycobacterium tuberculosis*.

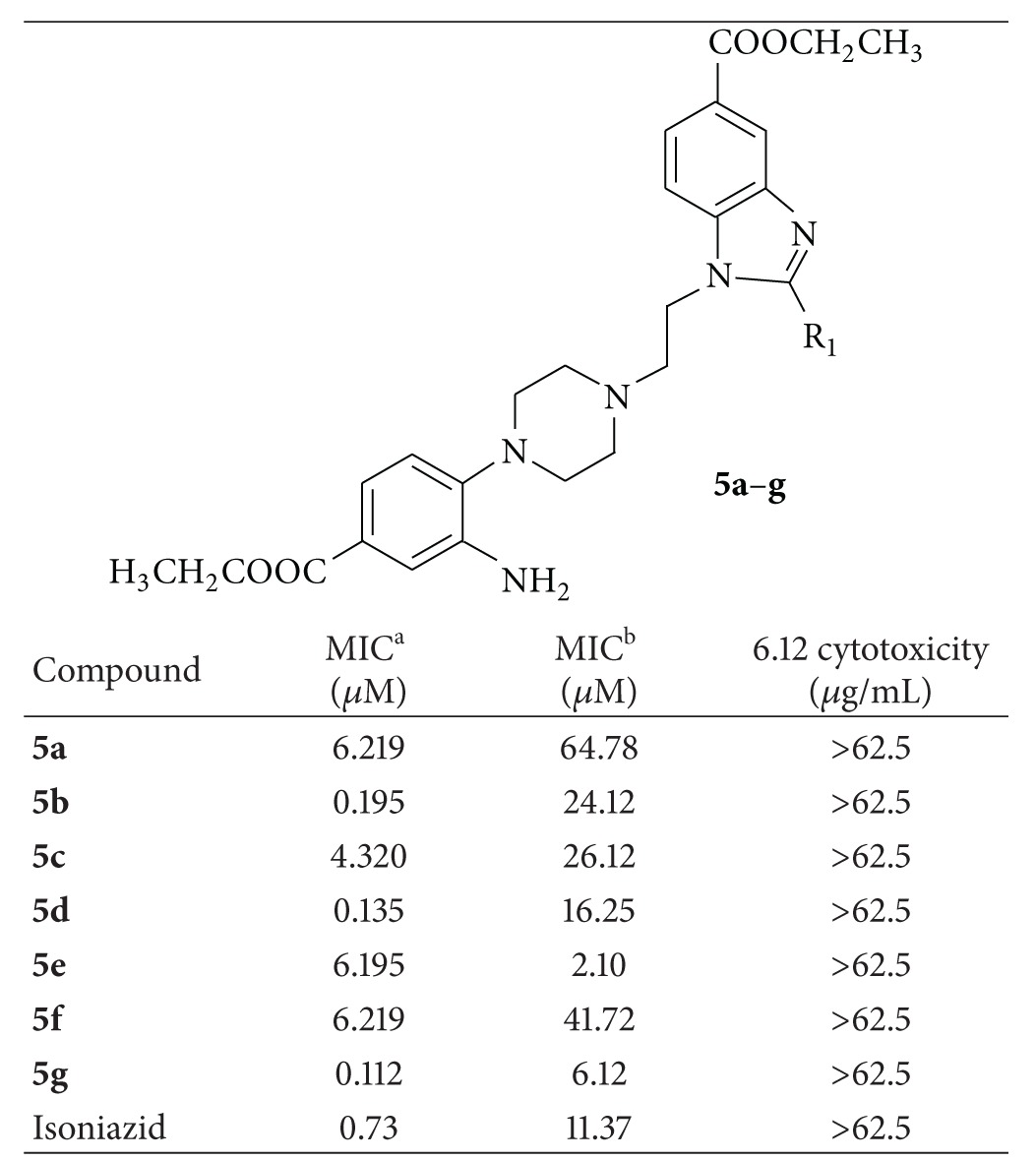

^a^ *Mycobacterium tuberculosis* H_37_Rv and ^b^INH-resistant *Mycobacterium tuberculosis*.
