# Supplementary material for: Antituberculosis: Synthesis and Antimycobacterial Activity of Novel Benzimidazole Derivatives
Source: Biomed Res Int. 2013 Dec 5;2013:926309. doi: 10.1155/2013/926309 (PMC3870127; doi:10.1155/2013/926309)
Supplement: Supplementary file 1 — S1: 1H NMR for compound 5d. S2: 13C NMR for compound 5d. S3: Direct infusion LC-MS for compound 5d. [file 926309.f1.pdf]

SUPPLEMENTARY MATERIALS

Antituberclosis :Syntheis and antimycobacterial activity of novel benzimidazole Derivatives

Yeong Keng Yoon[a], Mohamed Ashraf Ali[*,a,b,c], Tan Soo Choon[a], Rusli Ismail[e], Ang Chee Wei[a], Raju Suresh Kumar[d], Hasna Oshman[d], Farzana Beevi[c]

[a]*Institute for Research in Molecular Medicine, Universiti Sains Malaysia, Minden, 11800 Penang, Malaysia*

[b]*New Drug Discovery Research, Department of Medicinal Chemistry, Alwar Pharmacy College, Alwar, Rajasthan-301030, India*

[c]*New Drug Discovery Research, Department of Medicinal Chemistry, Sunrise University, Alwar, Rajasthan-301030, India*

[d]*School of Chemical Science, Universiti Sains Malaysia, Minden, 11800 Penang, Malaysia*

[e]*Centre of Excellence for Research in AIDS (CERiA), University Malaya, 50603 Kuala Lumpur, Malaysia.*

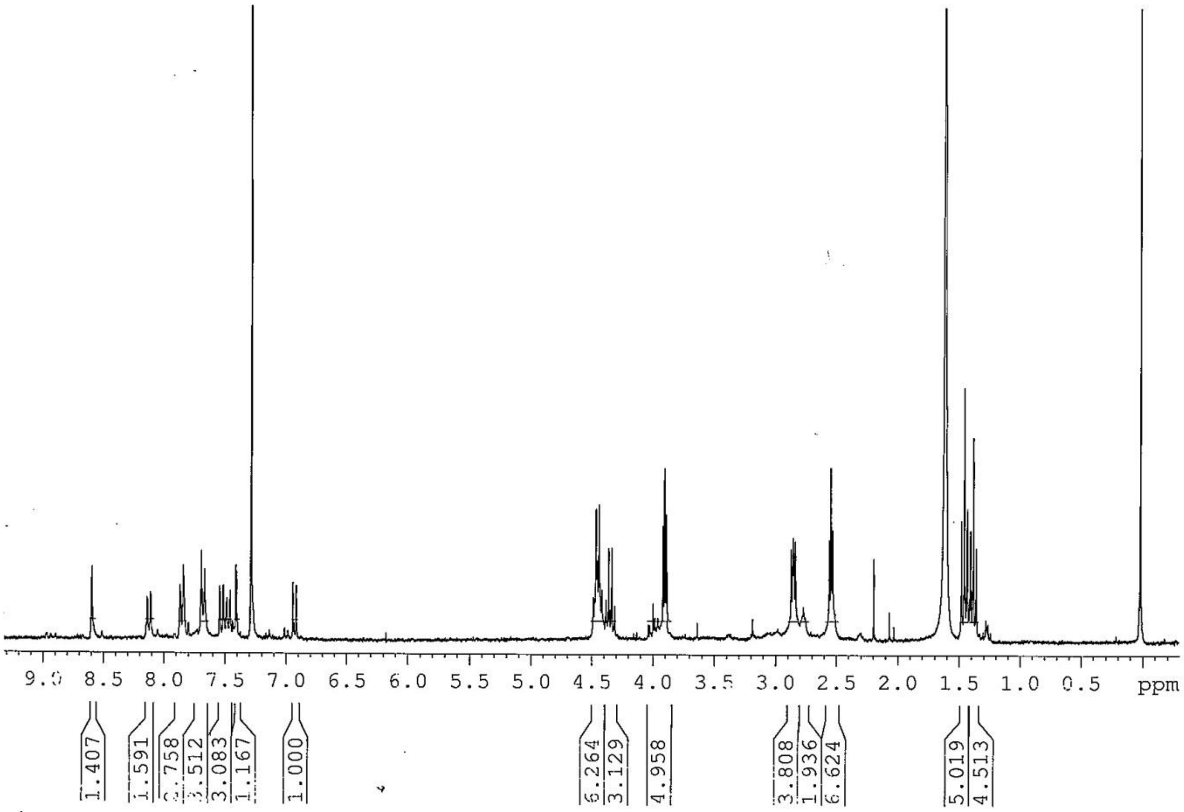

S1. ^{1}H NMR for compound **5d**

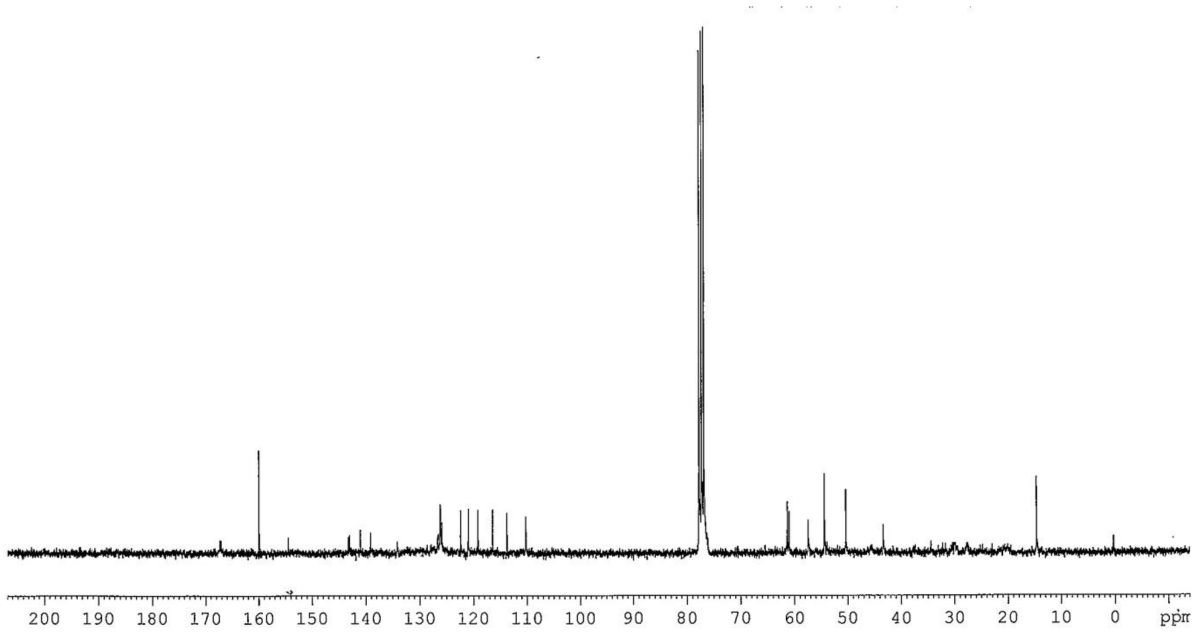

S2. ^{13}C NMR for compound **5d**

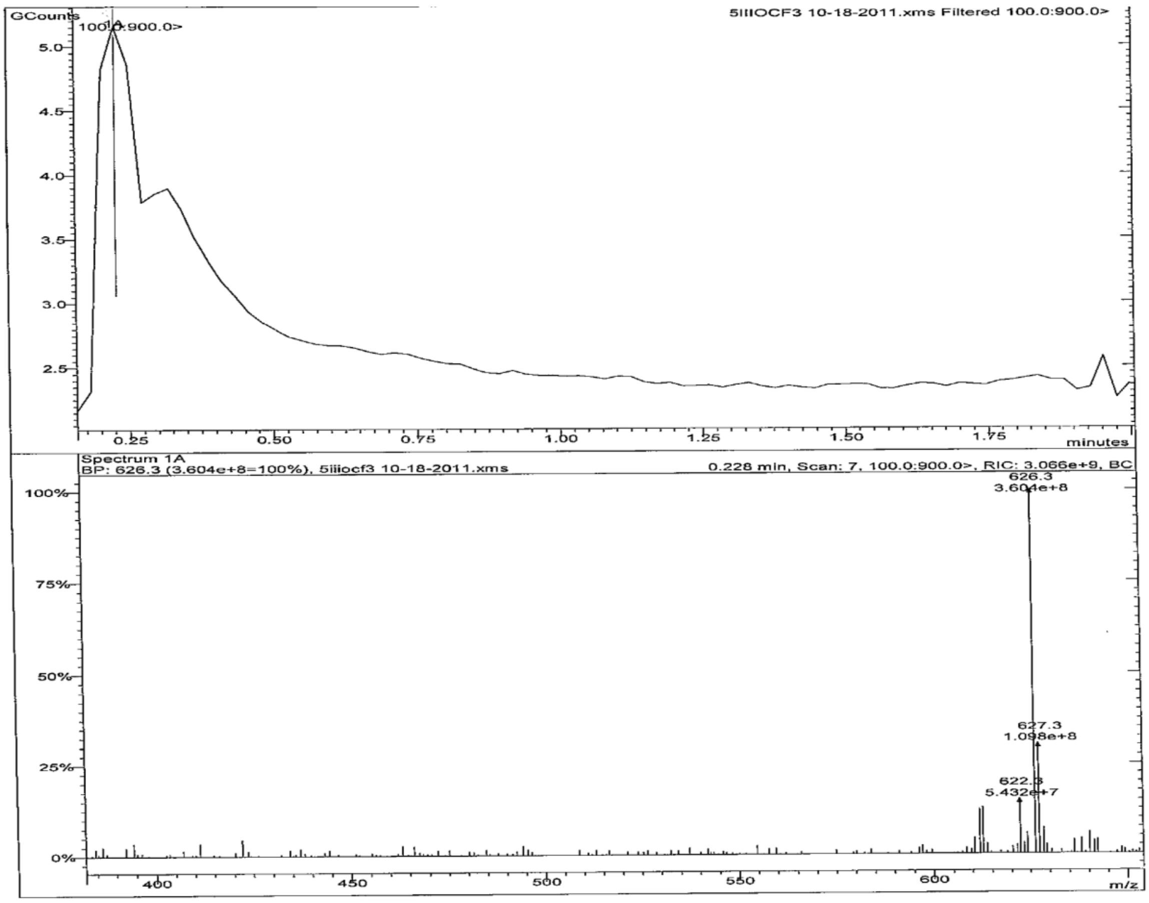

S3. Direct infusion LC-MS for compound **5d**
